# Phase II trial of sorafenib and doxorubicin in patients with advanced hepatocellular carcinoma after disease progression on sorafenib

**DOI:** 10.1002/cam4.3389

**Published:** 2020-08-25

**Authors:** Imane El Dika, Marinela Capanu, Joanne F. Chou, James J. Harding, Michele Ly, Anna D. Hrabovsky, Richard K.G. Do, Jinru Shia, Brittanie Millang, Jennifer Ma, Eileen M. O’Reilly, Ghassan K. Abou‐Alfa

**Affiliations:** ^1^ Memorial Sloan Kettering Cancer Center New York NY USA; ^2^ Weill Cornell College of Medicine New York NY USA; ^3^ Sidney Kimmel Medical College of Thomas Jefferson University Philadelphia PA USA; ^4^ Albert Einstein College of Medicine New York NY USA

**Keywords:** ASK‐1, doxorubicin, HCC, hepatocellular carcinoma, pERK, second line, sorafenib

## Abstract

**Background:**

Patients with advanced hepatocellular carcinoma (HCC) who received second line sorafenib plus doxorubicin following disease progression on sorafenib were shown retrospectively to have improved progression free survival (PFS) and overall survival (OS). Sorafenib plus doxorubicin combination may synergistically promote *ASK‐1* mediated apoptosis in cancer cells through *RAF‐1* inhibition. Thus, we conducted this phase II study of sorafenib and doxorubicin combination following progression on sorafenib.

**Methods:**

Patients with histologically confirmed advanced HCC, confirmed radiologic progression on sorafenib, Karnofsky performance status (KPS) ≥70%, and Child‐Pugh A liver cirrhosis were eligible. Patients received sorafenib 400 mg twice daily and doxorubicin 60 mg/m^2^ once every 3‐weeks. The primary endpoint was OS at 6 months (OS6). Secondary endpoints included safety, PFS, OS, response rate (RR) by RECIST 1.1. Additional endpoints included baseline and on‐treatment tumor *ASK‐1* and *pERK* expression levels by immunohistochemistry (IHC) and the correlation with PFS, RR, and OS.

**Results:**

Thirty patients were enrolled in the study, 86% were male, median age was 64 years. OS6 was 76.6% (95%CI: 57.2%‐88.1%). Median OS was 8.6 (95%CI: 7.3‐12) months, and median PFS reached 3.9 (95%CI: 2.4‐4.6) months. Three (11%) partial responses were observed and 17 patients (61%) had stable disease. Pertinent grade 3‐4 adverse events that occurred in more than 10% of patients included neutropenia (16%), febrile neutropenia (10%), anemia (10%), thrombocytopenia (10%), elevated AST (23%) and ALT (10%), hypophosphatemia (10%), and fatigue (10%). No association with the difference in baseline and post‐treatment *ASK‐1* and *pERK* level of expression by IHC and survival outcomes was detected.

**Conclusion:**

Sorafenib plus doxorubicin following progression on sorafenib did not show any improved outcome. We do not recommend further development or use of this combination in HCC.

## Graphical abstract

Sorafenib plus doxorubicin was evaluated as a second line therapy for advanced HCC. The association with the difference in baseline and post treatment ASK‐1 and pERK level of expression by IHC and survival outcomes was evaluated yet not detected. Sorafenib plus doxorubicin following progression on sorafenib did not show any improved outcome.

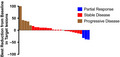

## 1 INTRODUCTION

At the time of this study conception and initiation, there was an imminent need for the identification of second line therapies in patients with advanced hepatocellular carcinoma (HCC). The combination of doxorubicin and sorafenib was initially evaluated as first line therapy in a multinational, phase II, randomized, double‐blind study. In this study, 96 patients with advanced HCC were randomized to doxorubicin plus sorafenib, and doxorubicin plus placebo, as sorafenib was not yet a proven standard of care.^1^ Median OS was 13.7 months (95% CI, 8.9‐‐not reached) for the doxorubicin sorafenib and 6.5 months (95% CI, 4.5‐9.9; *P* = .006) for doxorubicin alone. PFS was improved from 2.7 months (95% CI, 1.4‐2.8) to 6 months (95% CI, 4.6‐8.6) in the combination arm (*P* = .006). Given the favorable outcomes observed in the initial prospective phase II study, a retrospective analysis of 14 patients with HCC who received sorafenib and doxorubicin after progression on sorafenib demonstrated potential clinical activity. Five patients (36%) had stable disease (SD) for longer than 4 months duration. Median PFS was 3.4 months (95% CI 2.30‐8.36), and OS was 10.1 months (95% CI 4.34‐11.45).^2^ Based on the above, a national multicenter phase III randomized study of sorafenib vs sorafenib and doxorubicin in untreated patients with HCC (CALGB 80 802) followed.^3^ CALGB 80 802 was not reported at the time of this study conception. Ultimately, of 356 patients accrued on the study, the median OS was 9.3 months (95%CI 7.1‐12.9) for sorafenib and doxorubicin, and 10.5 months (95% CI 7.4‐14.3) for sorafenib monotherapy, hazard ratio (HR) 1.06 (95% CI 0.8‐1.4). Median PFS was 3.6 (95% CI 2.8‐4.6) and 3.2 months (95% CI 2.3‐4.1) respectively (HR = 0.90, 95% CI 0.72‐1.2). The addition of doxorubicin to sorafenib was associated with high toxicity and no improvement in OS or PFS outcomes.^3^, ^4^

Preclinical studies have shown that anthracycline cytotoxicity is mediated by the pro‐apoptotic apoptosis signal‐regulating kinase 1 (*ASK‐1*), one of the major *MAP3Ks*.^5^ *Raf‐1* binds to and neutralizes *ASK1*, which is required for endothelial cells apoptosis and chemotherapy cytotoxic effect, preventing the cells from undergoing apoptosis. Sorafenib inhibits *Raf‐1* which is expected to increase *ASK1* and tumor cells chemosensitivity to doxorubicin. This might elucidate a potential synergistic effect of both drugs.

Immunohistochemical study analysis of HCC tumors have demonstrated that mitogen‐activated protein kinase *(MAPK)* kinase/extracellular signal regulated kinase (*MEK/ERK*) signal transduction pathway has a significant role in the pathogenesis of HCC.^6^ In the phase II study of sorafenib for HCC,^7^ tumor cell staining for phosphorylated *ERK (pERK*) was correlated with time to disease progression. Patients with higher tumor cell *pERK* immunostaining intensity demonstrated a longer median time to progression (*P* = .00034, n = 15). These results suggested that higher expression levels of *pERK* at baseline was predictive of response to sorafenib therapy.

Thirdly, in vitro studies have shown that sorafenib appears to decrease the expression of multidrug resistance (MDR) protein gene, which leads to re‐sensitizing cells to certain chemotherapy such as gemcitabine and doxorubicin.^8^

Our study aimed to explore this potential synergy between sorafenib and doxorubicin. We also worked on characterizing the *pERK* and *ASK‐1* expression levels and immunostaining profiles in tumor samples obtained pre‐ and post‐treatment.

## 2 METHODS

This study was approved by the institutional review boards at Memorial Sloan Kettering Cancer Center and was registered at ClinicalTrials.gov (NCT 01840592). Written informed consent was obtained from all patients.

### 2.1 Patients

Patients 18 year of age or older diagnosed with histologically confirmed HCC were eligible. Eligible patients must have confirmed RECIST 1.1 radiologic progression on sorafenib, Eastern Cooperative Oncology Group (ECOG) performance status of 0 or 1 or KPS ≥ 70%, at most Child‐Pugh A liver cirrhosis, and adequate hematological, hepatic, cardiac, and renal function. Prior local therapies such as radiation or embolization were allowed except for doxorubicin drug eluting beads trans‐arterial chemo‐embolization, in fear of systemic release of doxorubicin and lack of understanding how to account of such exposure to cumulative lifetime exposure to doxorubicin. Patients with HBV or HCV infection were continued on antiviral therapy except for interferon.

### 2.2 Trial design

This was a nonrandomized, open label, single institution phase II study. Patients received sorafenib at 400 mg twice a day and doxorubicin 60 mg/m^2^ on first day of 3‐week cycle. Doses were adjusted according to baseline hepatic and renal function. For bilirubin up to 1.2 mg/dL within one week from Day 1 of Cycle 1 of therapy, patients received sorafenib at standard dose of 400 mg orally twice daily, or last tolerated dose from previous sorafenib based therapy, and doxorubicin 60 mg/m^2^ intravenously on Day 1 of each 3‐week cycle. For patients with bilirubin between 1.3 and 3 mg/dL within one week from start of therapy, sorafenib was administered at 400 mg orally once daily, or last tolerated dose from previous sorafenib based therapy, and doxorubicin 30 mg/m^2^ IV on first day of each cycle. Doxorubicin was administered for a maximum cumulative dose of 360 mg/m^2^. In circumstances of continued benefit and continued normal ejection fraction (≥50%) prior to each additional dose of doxorubicin, doxorubicin was allowed up to 450 mg/m^2^, followed by sorafenib given as a single agent. Treatment was continued until clinical or radiological disease progression, development of unacceptable toxicity, or withdrawal of content. A multigated acquisition (MUGA) scan or echocardiogram was performed every three cycles, up to the maximum cumulative dose of 360 mg/m^2^ of doxorubicin, then before every cycle thereafter up to the maximum total cumulative dose of 450 mg/m^2^. Continued normal ejection fraction (≥50%) was required before each additional dose of doxorubicin above 360 mg/m^2^.

### 2.3 Endpoints and assessments

Cross‐sectional imaging was obtained every three cycles. Responses were documented by Response Evaluation Criteria in Solid Tumors version 1.1 (RECIST 1.1).^9^ The primary endpoint was OS at 6 months. Secondary endpoints included PFS, OS, response rate by RECIST 1.1, and associations between duration of exposure to first line sorafenib and OS and PFS.

### 2.4 Correlative endpoints

Baseline and post‐treatment biopsy tissue samples were obtained and examined for correlative studies evaluating *pERK* and *ASK‐1* expression levels in tumor tissue. Post‐treatment biopsy was obtained after three cycles of treatment. The intensity of IHC staining (0+‐3+) and the percent of tumor cells staining (0%‐100%) for nuclear *pERK* IHC expression and cytoplasmic *ASK1* IHC expression were recorded by the study independent pathologist. A Histo (H) score which takes into account the intensity of staining and the percentage of tumor cells within the examined specimen, was generated for each stain.^10^

### 2.5 Statistical design

Simon's two‐stage minimax design was utilized with an unacceptable OS6 of 50%, an acceptable OS6 of 72%, a type I error of 5%, and type II error of 20%. Initially, 15 patients were to be enrolled in the first study stage. If seven or less patients are alive at 6 months, the trial was to be terminated, and if eight or more patients were alive at 6 months, an additional 15 patients were to be accrued for a total of 30 participants. If 20 or more of the 30 patients were alive at 6 months of treatment, then further investigation with a larger number of patients would be warranted. All evaluations were based on an intent‐to‐treat analysis. Patients who were removed from study were monitored for survival, unless consent for continued follow‐up was withdrawn. PFS was calculated from study entry to documented disease progression or death from any cause, whichever occurred first. OS was calculated from study entry to death or last follow‐up. PFS and OS were estimated using the Kaplan‐Meier methodology. The documented response rate and exact 95% confidence intervals (CI) were calculated. Toxicity rate was reported by category and grade of severity according to the NCI common toxicity criteria version 3.^11^ Among patients with pre‐ and post‐biopsy tissues available for analysis, the difference in the mean distributions of *pERK* and *ASK‐1* expression scores were evaluated using signed‐rank test.^12^, ^13^ The association with the change of *pERK* and *ASK‐1* expression levels with OS and PFS was performed using a landmark survival analysis^14^ with 9 weeks from treatment as a landmark time. Patients with follow‐up less than 9 weeks were excluded from the landmark analysis. All statistical analyses were performed using SAS Version 9.4 (SAS Institute, INC.). All *P*‐values were two‐sided. *P*‐values of < .05 were considered to indicate statistical significance.

## 3 RESULTS

### 3.1 Demographics

Thirty patients were enrolled during the period between April 2013 and June 2016. The majority of patients were male (86%) and the median age was 64 years (range 24‐82). Most patients had extrahepatic metastasis (86%), and 50% of patients had elevated alpha‐fetoprotein (AFP) level exceeding 400 ng/mL. Baseline demographics and clinical characteristics are summarized in Table 1. Median doses of doxorubicin and sorafenib received on study were 94 and 380 mg respectively.

**TABLE 1:** Patients characteristics

	N (%)
Median age (range)	65 (24‐82)
Male	26 (87%)
Female	4 (13%)
White	20 (66%)
Asian	6 (20%)
N/A	4 (13%)
KPS 70	2 (7%)
80	26 (86%)
90	2 (7%)
Child Pugh score A	30 (100%)
B	0
Locally advanced	8 (27%)
Metastatic	22 (73%)
Extrahepatic Disease	26 (86%)
Portal Vein Invasion Yes	10 (24%)
No	20 (66%)
AFP < 400 ng/mL	15 (50%)
≥400 ng/mL	15 (50%)
Hepatitis B	4 (13%)
Hepatitis C	9 (30%)
Hepatitis B & C	3 (10%)
NAFLD	8 (26%)
Alcoholic Cirrhosis	2 (6%)
None	4 (13%)

### 3.2 Outcomes

**FIGURE 1:**
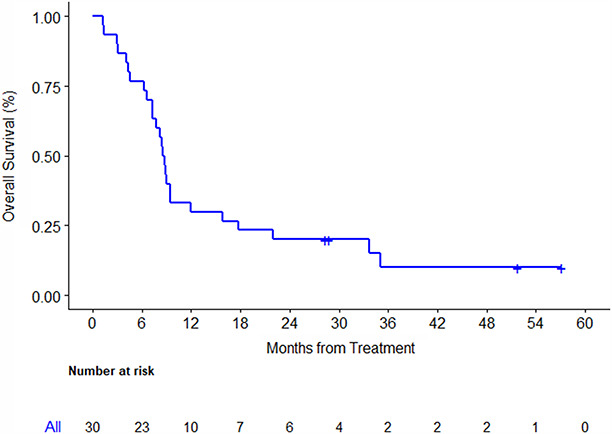
Overall survival

**FIGURE 2:**
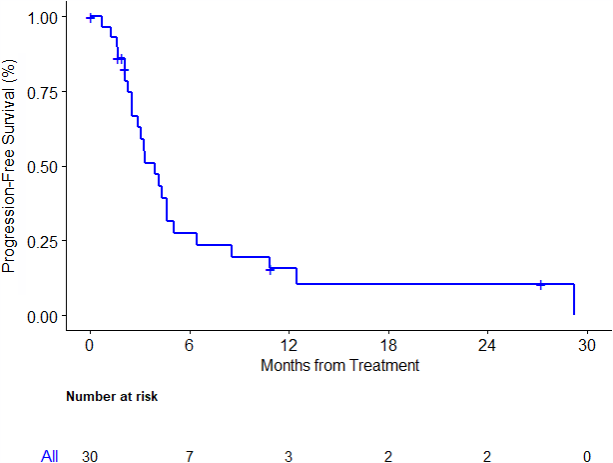
Progression free survival

**FIGURE 3:**
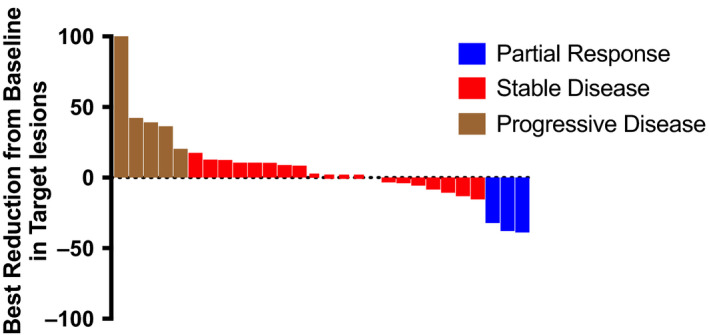
Response rates per RECIST 1.1

At the time of study analysis, median follow‐up among survivors was 40.2 [range: 28‐57] months. Twenty‐three patients were alive at 6 months, OS6 was 77% [95% (CI): 57.2%‐88.1%]. Median OS was 8.6 [95% CI: 7.3‐12] months (Figure 1), and PFS was 3.9 [95%CI: 2.5‐4.6] months (Figure 2). There were 3 (10.7% [95%CI: 2.2%‐28.2%]) partial responses and 17 (60.7% [95%CI: 40.5%‐78.4%) stable disease per RECIST1.1 (Figure 3) of 28 evaluable patients. Median duration of previous single agent sorafenib treatment was 3.3 [range: 0.9‐27] months. Comparing one patient who was treated 1 month longer on first line sorafenib to another, no association of either OS [HR 0.92 95%CI: 0.83‐1.02, *P* = .11] or PFS [HR 0.94 95%CI: 0.87‐1.02, *P* = .15] with 1 month increase of duration of exposure to first line sorafenib was detected.

### 3.3 Correlatives

Baseline *pERK* expression was positive in 9 of 13 cases as determined by IHC. Intensity was labeled as 0 (n = 4), 1+ (n = 2), 2+ (n = 2), and 3+ (n = 5). Post‐treatment *pERK* staining was positive in 9 of the 11 examined specimens. Intensity was 2+ (n = 1), and 3+ (n = 8). Baseline *ASK1* staining was evaluable in 13 samples, intensity was 0 (n = 6), 1+ (n = 5), and 2+ (n = 2). Post‐treatment *ASK1* intensity was 0 (n = 4), 1+ (n = 3), and 2+ (n = 4) in 11 evaluable samples. Matched pre‐ and post‐treatment tumor biopsies from 11 patients were available for the preplanned correlative analysis. We used Histo (H) score to take into consideration the percentage of tumor cells in the studied specimen. There was no difference in the distributions of *pERK* and *ASK‐1* before and after treatment (Table 2). In addition, there was no significant association between changes with *pERK* and *ASK‐1* markers and survival outcomes.

**TABLE 2:** Difference between pre‐ and post‐treatment p‐ERK and ASK1 stains

	Pre‐treatment H‐score [Mean (SD)]	Post‐treatment H‐ score [Mean (SD)]	Difference	*P*‐value
Difference (post‐pre)‐pERK score	0.17 (0.24)	0.54 (0.64)	0.34	.15
Difference (post‐pre) ASK Score	0.23 (0.34)	0.49 (0.55)	0.19	.35

### 3.4 Adverse events

All grade adverse events are detailed in Table 3. Pertinent grade 3‐4 adverse events occurring in more than 10% of patients included neutropenia (16%), fever and neutropenia (10%), anemia (10%), thrombocytopenia (10%), elevated AST (23%), elevated ALT (10%), hypophosphatemia (10%), and fatigue (13%). Two patients (6%) developed grade 2 decrease in left ventricular ejection fraction, and 1 (3%) patient developed grade 2 myocarditis. There were no deaths attributed to study treatment.

**TABLE 3:** Adverse events

Table 2: Adverse events	All Grades	G3,4, any relationship	G3,4, related
General			
Fatigue	28 (93%)	6 (20%)	4 (13%)
Nausea	20 (67%)	2 (7%)	2 (7%)
Anorexia	16 (53%)	0 (0%)	0 (0%)
Diarrhea	16 (53%)	2 (7%)	2 (7%)
Vomiting	10 (33%)	1 (3%)	1 (3%)
Mucositis	10 (33%)	2 (7%)	1 (3%)
Colitis	2 (7%)	0 (0%)	0 (0%)
Weight loss	9 (30%)	0 (0%)	0 (0%)
Edema	16 (53%)	2 (7%)	0 (0%)
Alopecia	12 (40%)	0 (0%)	0 (0%)
Palmar‐plantar erythrodysesthesia	10 (33%)	2 (7%)	2 (7%)
Confusion	5 (17%)	0 (0%)	0 (0%)
Hematological			
Neutropenia	10 (33%)	7 (23%)	7 (23%)
Febrile neutropenia	3 (10%)	3 (10%)	2 (7%)
Thrombocytopenia	24 (80%)	4 (13%)	4 (13%)
Anemia	7 (23%)	4 (13%)	3 (10%)
Increased INR	17 (57%)	1 (3%)	0 (0%)
Liver			
AST elevation	30 (100%)	9 (30%)	3 (10%)
ALK PHOS elevation	24 (80%)	1 (3%)	1 (3%)
ALT elevation	21 (70%)	3 (10%)	3 (10%)
Bilirubin elevation	15 (50%)	6 (20%)	1 (3%)
Low albumin	28 (93%)	0 (0%)	0 (0%)
Cardiac			
Decrease in LVEF	2 (6%)	0	0
Chest pain(myocarditis)	1 (3%)	0	0
Other			
Creatinine	5 (17%)	0 (0%)	0 (0%)
Ascites	10 (33%)	1 (3%)	0 (0%)
Increased lipase	16 (53%)	4 (13%)	2 (7%)
Increased amylase	8 (27%)	1 (3%)	0 (0%)
Hyperglycemia	29 (97%)	2 (7%)	0 (0%)

## 4 DISCUSSION

The addition of doxorubicin to sorafenib therapy after disease progression using front‐line sorafenib in patients in need for systemic therapy for HCC demonstrated an OS6 of 76.6% (95%CI: 57.2%‐88.1%). While we may claim that we met the prespecified threshold of activity, median OS and PFS were limited to 8.6 (95% CI: 7.3‐12) and 3.6 (95%CI: 2.4‐4.4) months respectively. This study was developed prior to the advent of all new systemic therapies in the second line setting,^15^, ^16^, ^17^, ^18^, ^19^ and in retrospect the combination fared worse compared to historical controls where OS in second line setting ranges around 9 months, in addition to the notable toxicity.^20^ The three patients with radiographic partial responses (RECIST 1.1) on the study are intriguing. One may wonder if these responses might be attributed to the doxorubicin and sorafenib combination or to the antiangiogenic effect of sorafenib after a brief interruption. Regardless we do not envision or support the use or to further evaluate the combination of sorafenib and doxorubicin.

*RAF‐1* binds to *ASK1* and may prevent apoptosis. Sorafenib, by inhibiting *RAF‐1* pathway, could promote apoptosis and sensitize HCC tumor cells to doxorubicin. This hypothesis was tested through the study correlatives herein. The baseline and on‐treatment tumor tissue staining for *pERK* and *ASK1* failed to confirm this hypothesis. There was no significant change in the biomarker expression after treatment with the combination or correlation between level of *ASK‐1* and *pERK* expression with clinical outcomes. It was speculated that adding doxorubicin to sorafenib would induce response by promoting apoptosis, however, there was no increase in *ASK1* marker of apoptosis^5^ after treatment to support this theory. Furthermore, longer exposure to sorafenib as determined by duration of sorafenib as first line is anticipated to cause more profound *RAF‐1* inhibition and increase the response to the combination, which was not demonstrated based on correlation of OS and PFS to duration of sorafenib, HR of 0.92 and 0.94 respectively. In our study, *pERK* levels did not correlate with response to treatment with sorafenib and doxorubicin, as previously shown with sorafenib alone.^7^

The study is limited by the small sample size, nonrandomized and open‐label design and the number of patients who declined post‐treatment biopsy. This did not permit any subgroup analysis based on any demographics. Tumor biopsy was suboptimal for evaluation in some cases due to low tumor content and low cellularity.

Doxorubicin, based on this study and recently reported CALGB 80802,^4^ proved to have limited activity in HCC. The current landscape of HCC treatment has shifted from cytotoxic chemotherapy to tyrosine kinase inhibitors^15^, ^16^, ^21^, ^22^ and checkpoint inhibitor therapy,^17^, ^18^ both providing positive outcomes as single agents or in combinations.

## CONFLICT OF INTEREST

Imane El Dika, Marinela Capanu, Joanne F. Chou, James J. Harding, Michele Ly, Anna D. Hrabovsky, Richard KG Do, Jinru Shia, Brittanie Millang, Jennifer Ma: (None), Eileen M. O’Reilly and Ghassan K. Abou‐Alfa: ActaBiologica, Agios, Array, Astra Zeneca, Bayer, Beigene, BMS, Casi, Celgene, Exelixis, Genentech, Halozyme, Incyte, Mabvax, Polaris Puma, QED, Roche(Research grants) Agios, Astra Zeneca, Autem, Bayer, Beigene, Berry Genomics, Bioline, Celgene, CytomX, Debio, Eisai, Eli Lilly, Flatiron, Genentech, Genoscience, Gilead, Incyte, Ipsen, LAM, Loxo, Merck, MINA, QED, Redhill, Roche, Silenseed, Sillajen, Sobi, Targovax, Therabionics, Twoxar, Yiviva (Consulting).

## AUTHOR CONTRIBUTIONS

**Conceptualization:** Ghassan K. Abou‐Alfa; **Data curation:** Imane El Dika, Marinela Capanu, Joanne F. Chou, Richard KG Do, Jinru Shia Ghassan K. Abou‐Alfa; **Formal analysis:** Imane El Dika, Marinela Capanu, Joanne F. Chou, Richard KG Do, Jinru Shia, and Ghassan K. Abou‐Alfa; **Funding acquisition:** Ghassan K. Abou‐Alfa; **Investigation:** Imane El Dika, Marinela Capanu, Joanne F. Chou, James J. Harding, Michele Ly, Anna D. Hrabovsky, Richard KG Do, Jinru Shia, Brittanie Millang, Jennifer Ma, Eileen M. O’Reilly, Ghassan K. Abou‐Alfa Methodology: Ghassan K. Abou‐Alfa; **Project administrator:** Brittanie Millang; **Resources:** Michele Ly, Anna D. Hrabovsky, Brittanie Millang, and Jennifer Ma; **Software:** Michele Ly, Anna D. Hrabovsky, Brittanie Millang, and Jennifer Ma; **Supervision:** Ghassan K. Abou‐Alfa; **Validation:** Imane El Dika, Marinela Capanu, Joanne F. Chou, Richard KG Do, Jinru Shia, and Ghassan K. Abou‐Alfa; **Funding acquisition:** Ghassan K. Abou‐Alfa; **Visualization:** Imane El Dika, Marinela Capanu, Eileen M. O’Reilly, and Ghassan K. Abou‐Alfa; **Writing original draft:** Imane El Dika and Ghassan K. Abou‐Alfa; **Writing review and editing:** Imane El Dika, Marinela Capanu, Joanne F. Chou, James J. Harding, Michele Ly, Anna D. Hrabovsky, Richard KG Do, Jinru Shia, Brittanie Millang, Jennifer Ma, Eileen M. O’Reilly, Ghassan K. Abou‐Alfa.

## Data Availability

The data that support the findings of this study are available on request from the corresponding author. The data are not publicly available due to privacy or ethical restrictions.

## References

[1] Abou‐Alfa GK , Johnson P , Knox JJ , et al. Doxorubicin plus sorafenib vs doxorubicin alone in patients with advanced hepatocellular carcinoma: a randomized trial. JAMA. 2010;304(19):2154‐2160. 21081728 10.1001/jama.2010.1672

[2] Abou‐Alfa GK , Ma J , O'Reilly EM , et al. Retrospective review of doxorubicin plus sorafenib as second‐line therapy in hepatocellular carcinoma (HCC). J Clin Oncol. 2012;30(4_suppl):298.

[3] Abou‐Alfa GK , Niedzwieski D , Knox JJ , et al. Phase III randomized study of sorafenib plus doxorubicin versus sorafenib in patients with advanced hepatocellular carcinoma (HCC): CALGB 80802 (Alliance). J Clin Oncol. 2016;34(4_suppl):192.

[4] Abou‐Alfa GK , Shi Q , Knox JJ , et al. Assessment of treatment with sorafenib plus doxorubicin vs sorafenib alone in patients with advanced hepatocellular carcinoma: phase 3 CALGB 80802 randomized clinical trial. JAMA Oncol. 2019;5(11):1582. 10.1001/jamaoncol.2019.2792 PMC6735405 31486832

[5] Alavi AS , Acevedo L , Min W , Cheresh DA . Chemoresistance of endothelial cells induced by basic fibroblast growth factor depends on Raf‐1‐mediated inhibition of the proapoptotic kinase, ASK1. Can Res. 2007;67(6):2766‐2772. 10.1158/0008-5472.CAN-06-3648 17363598

[6] Huynh H , Nguyen TT , Chow KH , Tan PH , Soo KC , Tran E . Over‐expression of the mitogen‐activated protein kinase (MAPK) kinase (MEK)‐MAPK in hepatocellular carcinoma: its role in tumor progression and apoptosis. BMC Gastroenterol. 2003;3:19. 12906713 10.1186/1471-230X-3-19 PMC317301

[7] Abou‐Alfa GK , Schwartz L , Ricci S , et al. Phase II study of sorafenib in patients with advanced hepatocellular carcinoma. J Clin Oncol. 2006;24(26):4293‐4300. 16908937 10.1200/JCO.2005.01.3441

[8] Hoffmann K , Franz C , Xiao Z , et al. Sorafenib modulates the gene expression of multi‐drug resistance mediating ATP‐binding cassette proteins in experimental hepatocellular carcinoma. Anticancer Res. 2010;30(11):4503‐4508. 21115899

[9] Eisenhauer EA , Therasse P , Bogaerts J , et al. New response evaluation criteria in solid tumours: Revised RECIST guideline (version 1.1). Eur J Cancer. 2009;45(2):228‐247. 19097774 10.1016/j.ejca.2008.10.026

[10] Thike AA , Chng MJ , Fook‐Chung S , Tan PH . Immunohistochemical expression of hormone receptors in invasive breast carcinoma: correlation of results of H‐score with pathological parameters. Pathology. 2001;33(1):21‐25. 11280603

[11] Trotti A , Colevas A , Setser A , et al. CTCAE v3.0: development of a comprehensive grading system for the adverse effects of cancer treatment. Seminars Radiation Oncol. 2003;13(3):176‐181. 10.1016/S1053-4296(03)00031-6 12903007

[12] Lehman EL . Nonparametrics: statistical methods based on ranks. San Francisco: Holden‐Day; 1975.

[13] MHaDAW . Nonparametric statistical methods. New York, NY: John Wiley & Sons; 1973:27‐33 (one‐sample), 68–75 (two‐sample).

[14] Anderson JR , Cain KC , Gelber RD . Analysis of survival by tumor response. J Clin Oncol. 1983;1(11):710‐719. 6668489 10.1200/JCO.1983.1.11.710

[15] Abou‐Alfa GK , Meyer T , Cheng A‐L , et al. Cabozantinib in patients with advanced and progressing hepatocellular carcinoma. New Engl J Med. 2018;379(1):54‐63. 29972759 10.1056/NEJMoa1717002 PMC7523244

[16] Bruix J , Qin S , Merle P , et al. Regorafenib for patients with hepatocellular carcinoma who progressed on sorafenib treatment (RESORCE): a randomised, double‐blind, placebo‐controlled, phase 3 trial. Lancet (London, England). 2017;389(10064):56‐66. 10.1016/S0140-6736(16)32453-9 27932229

[17] El‐Khoueiry AB , Sangro B , Yau T , et al. Nivolumab in patients with advanced hepatocellular carcinoma (CheckMate 040): an open‐label, non‐comparative, phase 1/2 dose escalation and expansion trial. Lancet (London, England). 2017;389(10088):2492‐2502. 10.1016/S0140-6736(17)31046-2 PMC7539326 28434648

[18] Zhu AX , Finn RS , Edeline J , et al. Pembrolizumab in patients with advanced hepatocellular carcinoma previously treated with sorafenib (KEYNOTE‐224): a non‐randomised, open‐label phase 2 trial. Lancet Oncol. 2018;19(7):940‐952. 29875066 10.1016/S1470-2045(18)30351-6

[19] Zhu AX , Kang YK , Yen CJ , et al. Ramucirumab after sorafenib in patients with advanced hepatocellular carcinoma and increased alpha‐fetoprotein concentrations (REACH‐2): a randomised, double‐blind, placebo‐controlled, phase 3 trial. Lancet Oncol. 2019;20(2):282‐296. 30665869 10.1016/S1470-2045(18)30937-9

[20] Zhu AX , Park JO , Ryoo B‐Y , et al. Ramucirumab versus placebo as second‐line treatment in patients with advanced hepatocellular carcinoma following first‐line therapy with sorafenib (REACH): a randomised, double‐blind, multicentre, phase 3 trial. Lancet Oncol. 2015;16(7):859‐870. 26095784 10.1016/S1470-2045(15)00050-9

[21] Llovet JM , Ricci S , Mazzaferro V , et al. Sorafenib in advanced hepatocellular carcinoma. New Engl J Med. 2008;359(4):378‐390. 18650514 10.1056/NEJMoa0708857

[22] Kudo M , Finn RS , Qin S , et al. Lenvatinib versus sorafenib in first‐line treatment of patients with unresectable hepatocellular carcinoma: a randomised phase 3 non‐inferiority trial. Lancet (London, England). 2018;391(10126):1163‐1173. 10.1016/S0140-6736(18)30207-1 29433850

